# Continuous Non-Invasive Monitoring of Hive Entrance Activity Reveals Honey Bee Colony Dynamics

**DOI:** 10.3390/biology15090731

**Published:** 2026-05-06

**Authors:** Cansu Özge Tozkar

**Affiliations:** Department of Agricultural Biotechnology, Faculty of Agriculture, Van Yüzüncü Yıl University, Van 65080, Turkey; tozkar@gmail.com

**Keywords:** automated monitoring, behavioral ecology, foraging behavior, hive entrance activity, honey bee colony health, precision apiculture

## Abstract

Honey bees are essential pollinators and ecological indicators, yet conventional monitoring approaches remain labor-intensive and limit the assessment of colony dynamics across biologically relevant time scales. Hive entrance activity, defined as the movement of bees entering and leaving the hive, serves as a direct proxy for foraging effort, resource availability, and colony condition. However, manual observation is restricted in duration and resolution, constraining continuous evaluation of colony responses to environmental change. In this study, a non-invasive automated monitoring framework was developed to quantify hive entrance activity under diverse environmental conditions. The system integrates image-based detection, multi-object tracking, and direction-aware trajectory analysis to identify and quantify incoming and outgoing bees. Trained on 12,013 annotated images representing varied lighting conditions, backgrounds, and activity levels, the framework achieved high detection accuracy (mAP@50 = 0.96–0.98) and real-time processing capability. Video-based evaluations showed that frame-level detections can be translated into entrance and exit estimates, although counting accuracy varied with traffic density and occlusion. These findings demonstrate that automated monitoring enables continuous measurement of biologically meaningful indicators of colony dynamics. The proposed approach provides a scalable tool for investigating foraging activity, seasonal patterns, and colony responses to environmental stressors, supporting improved ecological assessment and pollinator conservation.

## Simple Summary

Monitoring bee colony health typically requires opening hives or maintaining human observers at the entrance for extended periods, both of which are disruptive and impractical at scale. This study describes a camera-based system that watches the hive entrance automatically, counts bees going in and out, and does so without disturbing the colony. Using artificial intelligence trained on over 12,013 images, the system can identify individual bees in real time and follow their movements to determine whether each bee is entering or leaving the hive. Tests on video recordings showed that the system works well under low traffic conditions, though accuracy drops when many bees move simultaneously. The approach is practical, low-cost, and scalable to multiple colonies, making it a useful tool for beekeepers and researchers who want to track colony activity continuously without manual effort.

## 1. Introduction

Pollinating insects underpin the reproductive success of approximately 87% of flowering plant species and contribute substantially to global food production through crop pollination [1,2]. Among managed pollinators, the Western honey bee (*Apis mellifera* L.) represents the most economically significant species, providing pollination services valued at billions of dollars annually [3]. Beyond their agricultural importance, honey bee colonies serve as powerful model systems for studying social insect biology, collective decision-making, and population-level responses to environmental disturbance [4,5,6]. Yet despite their ecological prominence and economic value, honey bee populations face multifaceted threats, including habitat loss, pesticide exposure, pathogens, parasites, and climate-induced phenological mismatches [7,8,9].

Effective conservation and management strategies require robust monitoring systems capable of detecting population declines and identifying causal mechanisms [10]. Honey bee colony health is best assessed using colony-level metrics and integrated indicators that reflect the dynamics and resilience of the super-organism as a whole, rather than metrics focused solely on individual workers [11]. Among measurable colony traits, the rate of forager departures and returns at hive entrances directly reflects colony foraging effort, workforce availability, and resource acquisition dynamics [12,13]. Entrance activity patterns correlate with colony size, brood production, food stores, and external resource availability, making traffic counts a valuable non-invasive assessment tool [12,14,15]. Seasonal and diurnal fluctuations in bee activity at and around the hive entrance reflect colony responses to temperature, photoperiod, and floral resource phenology [16,17].

Traditional methods for quantifying hive entrance activity rely on direct human observation, typically involving timed counts of passing individuals at hive [15,18]. While conceptually straightforward, this approach suffers from severe practical limitations. Manual counting of hive entrance activity is labor-intensive and typically constrained to brief observation intervals, limiting its ability to capture biologically meaningful variation over hours, days, or seasons [19]. Observer fatigue and human-related limitations can introduce systematic errors in manual flight activity counts, particularly during periods of high traffic [20]. Distinguishing directional movement, which is critical for separating incoming from outgoing individuals, becomes increasingly difficult as activity intensifies. Importantly, human presence near hive entrances may itself alter bee behavior, introducing observer effects into datasets [21,22]. These constraints have historically limited entrance activity studies to small sample sizes, brief observation windows, and qualitative rather than quantitative analyses.

Recent technological advances offer potential solutions to these limitations. Electronic counters employing infrared sensors or radiofrequency identification (RFID) tags have enabled automated traffic monitoring, yet these systems require physical modifications to hive structures and may interfere with natural bee movement patterns [23]. Sensor-based approaches further struggle to distinguish between incoming and outgoing individuals without directional gating mechanisms, which themselves constrain bee passage and introduce artifacts [24]. An ideal monitoring system would operate non-invasively, distinguish directional movement, handle variable traffic densities, function across diverse environmental conditions, and scale to multi-colony or population-level deployments.

Computer vision and deep learning technologies present promising alternatives for automated insect monitoring [25]. Object detection algorithms trained on annotated image datasets can identify and localize target organisms within video streams, enabling automated counts without physical intervention [26,27,28]. For small, fast-moving targets like flying insects, modern deep learning architectures such as YOLO (You Only Look Once) offer real-time detection capabilities with accuracy approaching or exceeding human performance [28,29]. When coupled with tracking algorithms that maintain individual identities across video frames, these systems can quantify directional movement, measure activity rates, and characterize behavioral patterns at scales unattainable through manual observation [27].

Despite growing interest in automated insect monitoring, most existing systems have primarily targeted general biodiversity assessment or agricultural pest detection, with comparatively limited emphasis on colony-level behavioral studies [25]. Applications to honey bees specifically have remained relatively limited, with few systems validated for sustained, field-realistic monitoring of entrance activity under diverse environmental conditions [30]. Key technical challenges include maintaining detection accuracy under variable illumination, distinguishing individual bees in high-density aggregations, tracking fast-moving targets across cluttered backgrounds, and achieving computational efficiency sufficient for real-time or long-term deployment [27].

Here, we address these challenges by developing and evaluating an automated system for detecting, tracking, and counting honey bee traffic at hive entrances. Our approach integrates state-of-the-art deep learning object detection with multi-object tracking algorithms designed for monitoring directional movement at defined spatial thresholds. We trained and compared multiple detection model architectures on an annotated image dataset spanning varied lighting conditions, backgrounds, and activity densities to ensure robust detection performance under realistic field conditions. We then assessed system-level counting performance through comparisons with ground-truth manual counts derived from independent video samples representing different traffic volumes. Our objectives were threefold: first, to evaluate whether automated image analysis can achieve high detection accuracy for individual honey bees at hive entrances; second, to compare detection performance and computational efficiency across multiple model architectures; and third, to demonstrate the feasibility of translating frame-level detections into video-level entrance and exit counts as a foundation for continuous, non-invasive colony monitoring. By establishing the technical basis for scalable assessment of entrance activity patterns, such tools promise to advance our understanding of honey bee foraging ecology, colony phenology, and population responses to environmental change. The remainder of this paper is organized as follows. Section 2 describes the image dataset construction, model architectures and training procedures, and the video-based counting pipeline. Section 3 presents image-level detection results across all evaluated models and video-level counting performance for the integrated system. Section 4 discusses the ecological implications of the findings, system limitations, and directions for future work. Section 5 provides concluding remarks.

## 2. Materials and Methods

### 2.1. Image Dataset Construction

The image data used in this study were obtained from multiple open-access honey bee detection datasets available on the Roboflow Universe platform [31]. To ensure relevance to the intended application of hive entrance traffic monitoring, only datasets containing images captured at hive entrances or landing boards were selected. Datasets depicting general foraging scenes, aerial views, or non-entrance contexts were excluded. All selected images were annotated with bounding boxes identifying individual honey bees. Since the images were sourced from multiple open-access datasets on the Roboflow Universe platform, pre-existing annotations were reviewed and harmonized according to a unified annotation guideline to ensure consistency across sources. Bounding boxes were drawn to tightly enclose the visible body of each bee, including partially visible individuals at frame boundaries, while excluding motion-blurred detections that could not be reliably localized. Images were resized to a fixed input resolution of 640 × 640 pixels. Standard data augmentation techniques supported by modern YOLO-based detection frameworks, including mosaic augmentation and color-space perturbations, were applied exclusively to the 8586 training images to improve robustness to moderate variations in scale, illumination, and background [32,33]. Augmentation was applied online during training via the Ultralytics framework’s built-in pipeline and was not applied to validation or test images. After data cleaning and deduplication, the final image dataset consisted of a total of 12,013 annotated frames. Of these, 8586 images were allocated for model training, forming the primary basis for learning discriminative visual features of honey bees at hive entrances. A further 2207 images were reserved for validation and used exclusively to monitor training convergence, guide model selection, and prevent overfitting. In addition, an independent test set comprising 1220 images was constructed and kept completely separate from the training and validation processes, serving solely for the final evaluation of detection performance on previously unseen data drawn from the same operational domain. The dataset split was designed to support domain-specific model optimization rather than cross-domain generalization. Since the dataset was compiled from multiple open-access sources, some degree of visual similarity between frames originating from the same recording sessions may exist across subsets. However, the use of multiple independent source datasets and the separation of training, validation, and test sets at the dataset level rather than at the frame level is expected to mitigate the impact of potential data leakage on the reported performance estimates.

**Figure 1:**
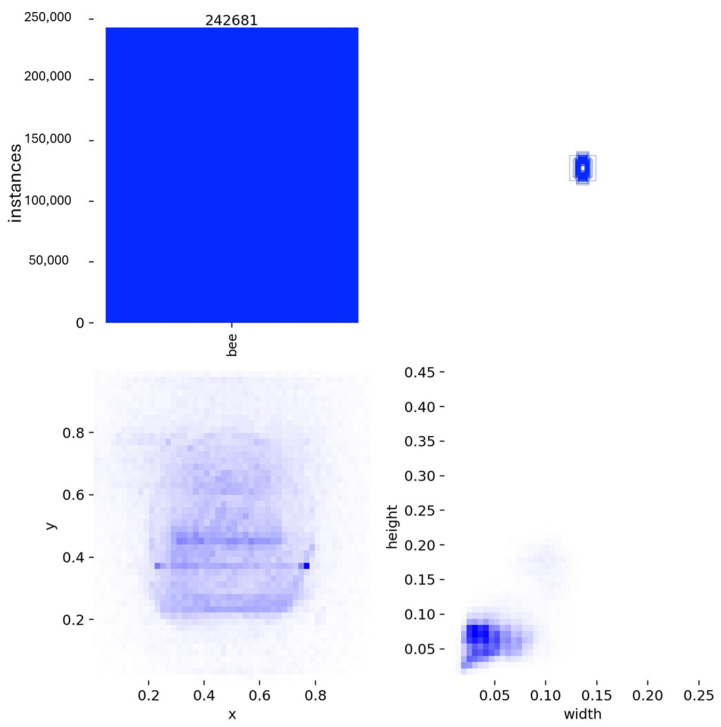
Dataset annotation statistics and spatial distribution of honey bee bounding boxes.

An analysis of the annotation statistics is presented in Figure 1. The distribution of bounding box centers across normalized image coordinates indicates a moderate central concentration, consistent with the typical framing of hive entrance imagery, without evidence of strong positional bias. The relative width and height distributions of bounding boxes show a compact and consistent range, reflecting the expected scale of individual honey bees within the image frames. Overall, these patterns indicate a well-structured and spatially balanced annotation set suitable for robust object detector training.

In addition to the image-level test set, video-based evaluation was conducted using a completely separate set of publicly available hive entrance videos that were not used during any stage of model training, validation, or image-based testing. This ensured a clear separation between image-level and system-level evaluation data.

### 2.2. Model Architecture and Training

Model training was performed on a CUDA-enabled desktop workstation equipped with an NVIDIA GeForce RTX 4070 SUPER GPU (12.9 GB VRAM; NVIDIA Corporation, Santa Clara, CA, USA), 64 GB system memory, and an Intel Core i7-14700K CPU (Intel Corporation, Santa Clara, CA, USA). The software environment consisted of Python 3.10 (v3.10), PyTorch 3.11.14 with CUDA support, and the Ultralytics YOLO framework (v8.3.3) [33]. Object detection models were trained using architectures from the YOLO family of one-stage object detectors [32,34]. Three architecture families were evaluated: YOLOv8 [33], YOLOv11 [35], and YOLOv26 [36]. For each family, two model variants differing in capacity were compared, namely the lightweight “n” (nano) variant and the larger “s” (small) variant, resulting in a total of six models (YOLOv8n, YOLOv8s, YOLOv11n, YOLOv11s, YOLOv26n, YOLOv26s). This design enables systematic examination of the trade-off between model complexity and detection performance both within and across architecture families. Pretrained weights provided by the Ultralytics framework were used to initialize the networks, enabling transfer learning and faster convergence. All models were trained using an input image resolution of 640 × 640 pixels. Training was conducted for a maximum of 100 epochs with a batch size of 16, while early stopping was enabled with a patience of 20 epochs to prevent overfitting. Optimization parameters were automatically determined within the framework, resulting in the use of stochastic gradient descent (SGD) with momentum. Model performance during training was monitored using standard detection metrics, including mean Average Precision at an Intersection over Union threshold of 0.5 (mAP@0.5), precision–recall curves, and F1-score. Final model weights were selected based on validation performance.

### 2.3. Video-Based Entrance–Exit Counting and Ground Truth

System-level evaluation was conducted using independent hive entrance videos obtained from publicly available online sources, which were not used during training, validation, or image-based testing. A region of interest (ROI) was manually defined around the hive entrance area using either polygonal or circular configurations, depending on entrance geometry. Bee trajectories were obtained using a tracking-by-detection approach based on the BoT-SORT multi-object tracking algorithm [37], applied to detection outputs generated by the YOLOv8 models. Although image-level detection performance was evaluated across six models from three architecture families, video-based counting experiments were conducted using only YOLOv8n and YOLOv8s, as these two variants provided representative trade-offs between computational efficiency and detection accuracy sufficient to demonstrate the feasibility of the proposed counting approach. Each detected bee was represented by its bounding box centroid, and trajectories were constructed by associating detections across consecutive frames. Entrance and exit events were identified using direction-aware crossings of the ROI boundary. System-level performance was evaluated using aggregate entrance and exit counts computed for each independent hive entrance video, with each video treated as a single evaluation unit. Automated counts were compared against manually established reference counts to assess counting accuracy at the video level. Counting accuracy was calculated as:

Accuracy (%) = [1 − (|*C_pred* − *C_gt|/C_gt*)] × 100,

where *C_pred* is the predicted count and *C_gt* is the manually determined ground-truth count. Using this formulation, an accuracy of 100% indicates perfect agreement between the automated count and the manually established reference count, whereas lower values indicate greater deviation from the ground truth.

Due to the inherent difficulty of establishing reliable ground truth under high traffic conditions, the evaluation focused on short video segments with relatively low traffic density in order to demonstrate the feasibility of the proposed counting approach. To enable reliable manual annotation, videos were temporally slowed down during the annotation process. Original video segments of approximately 15 s were replayed at reduced speed, corresponding to an effective duration of approximately 180 s. This procedure allowed accurate identification of entrance and exit events despite rapid bee movement near the hive entrance. By separating image-level detection evaluation from video-based counting assessment, the proposed framework provides a realistic representation of system behavior under practical hive monitoring conditions, where temporal continuity and trajectory consistency are essential.

## 3. Results

### 3.1. Image-Level Detection Performance

The image-level detection performance of the YOLO-based models was first evaluated to assess their ability to localize and identify individual honey bees at hive entrances under domain-specific conditions. This evaluation was conducted at the frame level and served to verify that the models successfully learned discriminative visual features relevant to the target application prior to system-level analysis.

On the validation dataset, all evaluated models achieved high detection accuracy, indicating effective convergence and stable learning behavior. Among the YOLOv8 variants, YOLOv8n achieved a mean Average Precision at an Intersection over Union threshold of 0.5 (mAP@50) of 0.98, with corresponding precision and recall values of 0.96 and 0.95, respectively. These results demonstrate that the lightweight YOLOv8n architecture provides reliable detection performance while maintaining low computational complexity. The larger YOLOv8s model exhibited slightly higher detection accuracy, achieving improved precision (0.97), recall (0.96), and mAP@50 (0.99), reflecting the expected trade-off between model capacity and performance.

Comparable trends were observed for the YOLOv11 and YOLOv26 architectures. For both model families, the “s” variants consistently outperformed their lightweight counterparts in terms of precision, recall, and mAP metrics, albeit at the cost of increased model size and training time. Overall, validation results indicate that all evaluated models are capable of robust honey bee detection under hive entrance conditions. A detailed summary of validation detection performance and computational characteristics is provided in Table 1. Detection metrics were computed at the best-performing epoch for each model.

Image-based test performance was evaluated using a fully independent test dataset composed of previously unseen frames from the same operational domain. As shown in Table 2, all models maintained high precision and recall on the test dataset, with mAP@50 values exceeding 0.96 across architectures. YOLOv8s achieved the highest overall test performance among the evaluated models, while also demonstrating efficient inference speed. Lightweight models such as YOLOv8n and YOLOv11n provided competitive accuracy with reduced computational cost, highlighting their suitability for resource-constrained deployments. Inference speed ranged from approximately 65 to 118 frames per second, confirming that all models are capable of near real-time image-level processing.

### 3.2. Comparison of YOLOv8n and YOLOv8s Models

**Figure 2:**
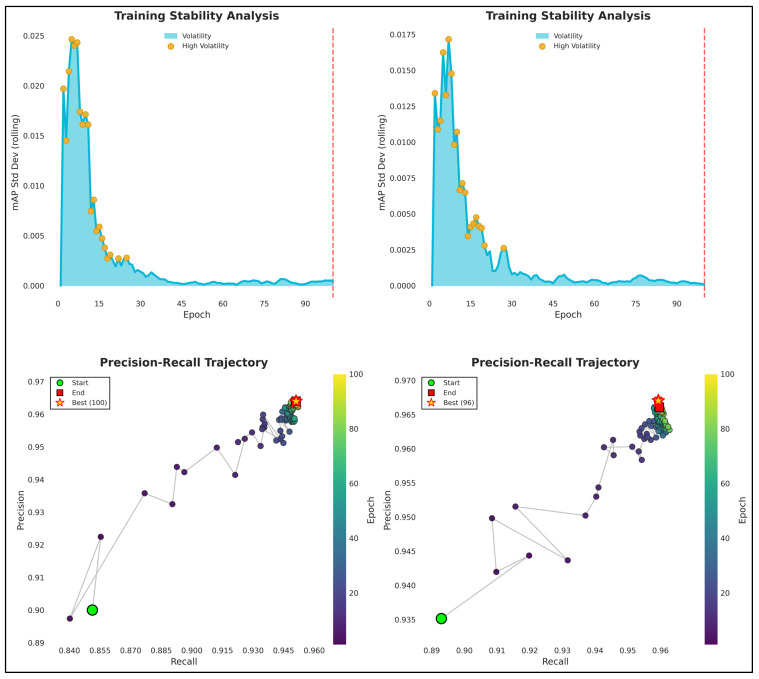
Training dynamics of YOLOv8n (**left**) and YOLOv8s (**right**), showing loss curves and detection metrics over training epochs.

A comparative analysis of YOLOv8n and YOLOv8s was conducted to examine the trade-off between model complexity, detection accuracy, and computational efficiency. Although YOLOv8s consistently outperformed YOLOv8n in image-level detection metrics, the observed performance gains were moderate relative to the increase in model size and computational cost. YOLOv8n provides a lightweight baseline that enables efficient inference with reduced computational requirements, making it suitable for resource-constrained deployments. In contrast, YOLOv8s offers improved detection robustness and higher accuracy, which can be advantageous under more challenging visual conditions. Importantly, this comparison highlights that image-level detection accuracy alone is insufficient to fully characterize system performance for entrance–exit counting tasks, where tracking stability and temporal trajectory continuity play a critical role. Training convergence behavior and learning dynamics for both YOLOv8 variants are illustrated in Figure 2. Both models exhibited stable convergence without signs of overfitting, confirming their suitability for subsequent system-level evaluation.

### 3.3. Video-Based Entrance–Exit Counting Performance

Based on the image-level detection accuracy and computational efficiency reported in Table 1 and Table 2, YOLOv8n and YOLOv8s were selected for video-based entrance–exit counting experiments. These two models represent complementary trade-offs between lightweight inference efficiency (YOLOv8n) and enhanced detection robustness (YOLOv8s). Other evaluated model variants did not provide additional methodological insight at the system level and were therefore excluded from further video-based analysis. Using the integrated detection, multi-object tracking, and ROI-based trajectory analysis pipeline, the proposed system successfully quantified aggregate entrance and exit counts for all evaluated hive entrance videos (Table 3). Both YOLOv8n and YOLOv8s enabled stable tracking of individual bees, allowing reliable identification of entrance and exit events based on direction-aware trajectory crossings. Quantitative comparison with manually established reference counts indicated that YOLOv8s generally achieved comparable or moderately improved counting accuracy relative to YOLOv8n, with more consistent performance observed for exit events. In some scenarios, YOLOv8s showed improved robustness involving brief occlusions and rapid bee movements near the hive entrance. However, both models exhibited notable counting deviations under higher traffic densities, and neither model consistently outperformed the other across all evaluated videos and event types. Given the limited duration of the evaluated videos and the relatively low number of counted events, the reported results should be interpreted as a proof-of-feasibility rather than a comprehensive large-scale validation. It should also be noted that the test videos used for counting evaluation were obtained from publicly available online sources with diverse and uncontrolled recording conditions, rather than from a standardized acquisition setup designed specifically for hive entrance monitoring. This heterogeneity in camera angle, resolution, distance, and background likely contributed to the observed variability in counting accuracy. Nevertheless, the findings demonstrate that the proposed pipeline can effectively translate frame-level detections into accurate video-level entrance–exit estimates under domain-specific conditions.

**Figure 3:**
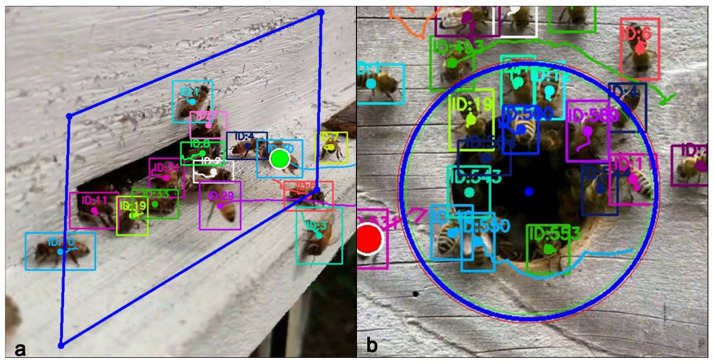
Example configurations of the region of interest (ROI) used for hive entrance monitoring and trajectory-based counting. (**a**) Polygonal ROI applied to a horizontal hive entrance, illustrating tracked bee identities and direction-aware boundary crossings. (**b**) Circular ROI configuration used for a vertical hive entrance, demonstrating stable multi-object tracking and aggregation of entrance and exit events.

A qualitative illustration of the detection, tracking, and counting pipeline is provided in Figure 3, demonstrating how individual bee trajectories are maintained across frames and how direction-aware crossings of the region of interest (ROI) boundary are used to identify entrance and exit events at the hive entrance.

**Figure 4:**
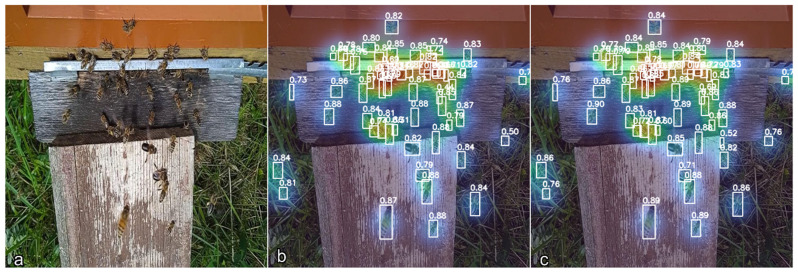
Qualitative visualization of model attention using Gradient-weighted Class Activation Mapping (Grad-CAM): (**a**) Original input frame with overlaid attention heatmap, (**b**) Grad-CAM response for YOLOv8n and (**c**) Grad-CAM response for YOLOv8s.

To provide additional qualitative insight into the visual cues used by the detection models, Grad-CAM visualizations were generated for representative frames. As illustrated in Figure 4, both YOLOv8n and YOLOv8s primarily attend to the body contours of individual bees, supporting the interpretability of the learned features under the evaluated conditions. The visualizations indicate that both models focus primarily on bee body structures during detection.

The limited number of evaluated videos was primarily due to the lack of standardized, high-quality, publicly available datasets suitable for direction-aware hive entrance monitoring, highlighting a current gap in the literature.

## 4. Discussion

Our findings show that deep learning–based object detection models can reliably identify individual honey bees at hive entrances under a wide range of visual conditions, achieving high image-level detection accuracy (mAP@50 = 0.96–0.98) on an independent test dataset. When combined with BoT-SORT multi-object tracking and direction-aware region-of-interest analysis, these models enabled automated quantification of entrance and exit events from video recordings. Although counting accuracy at the video level varied across evaluation scenarios, the overall results demonstrate the feasibility of automated, non-invasive monitoring of colony entrance activity. By avoiding limitations associated with observer fatigue, short observation periods, and potential human disturbance at the hive entrance, such systems provide new opportunities to investigate honey bee ecology, foraging dynamics, and colony responses to environmental change.

The results of this study provide a technical foundation upon which the following ecological applications can be realized. Although these applications have not been empirically tested within the scope of the present work, they represent the primary scientific motivation for developing automated entrance monitoring systems and are discussed here to contextualize the significance of the reported findings. From an ecological perspective, the capacity to quantify entrance traffic continuously across hours, days, and seasons represents a fundamental advance in colony-level monitoring. Entrance activity directly reflects colony foraging effort, which in turn depends on worker population size, brood production demands, food store levels, and external resource availability [38,39]. Temporal patterns in entrance traffic provide integrative measures of colony condition that respond to multiple internal and external drivers [13,40]. Previous studies using manual observation or limited sensor arrays (e.g., environmental sensors or simple optical counters) have documented diurnal and seasonal variation in entrance activity, revealing peaks coinciding with favorable temperature and floral resource availability [41,42,43]. However, these studies typically captured only brief snapshots of activity, limiting our understanding of how colonies adjust foraging effort in response to rapidly changing environmental conditions or resource landscapes.

Continuous monitoring enabled by automated systems allows investigation of colony responses to environmental drivers at ecologically relevant time scales. For example, minute-to-minute fluctuations in entrance traffic may reflect real-time adjustments to weather conditions, while hourly patterns reveal temperature- and light-dependent activity thresholds [44]. Day-to-day variation can indicate shifts in floral resource availability or colony nutritional demands, and seasonal trajectories document colony phenology, including spring buildup, summer foraging peaks, and autumn preparation for overwintering [12,45]. By capturing this hierarchy of temporal variation without the need for continuous human presence, automated monitoring systems generate datasets of exceptional temporal depth, enabling robust tests of hypotheses related to colony decision-making, foraging strategies, and adaptive responses to environmental heterogeneity.

Directional movement classification, that is, the distinction between incoming and outgoing bees, adds critical functional information to raw activity counts. Incoming traffic dominated by pollen-laden foragers indicates active resource collection, whereas more balanced bidirectional traffic can reflect orientation flights, robbing behavior, or other non-foraging activities [46,47]. Asymmetries between incoming and outgoing counts can signal unusual colony states, such as defensive behavior with net emigration, or conversely, abnormal retention of foragers due to navigational disruption [46]. Moreover, the ratio of pollen-bearing to non-pollen-bearing incoming foragers provides additional information about resource preferences and floral community composition [48], although the system developed in the present study does not yet distinguish pollen loads. Future integration of additional image analysis modules could extract such fine-grained behavioral data from entrance monitoring footage.

The image-level performance of the evaluated models across varied visual conditions, including different lighting regimes, backgrounds, and activity densities, addresses a critical requirement for field deployment in ecological research and apicultural practice. Natural hive entrances experience dramatic fluctuations in illumination across daily and seasonal cycles, from direct morning sunlight to shaded afternoon conditions and dawn/dusk transitions [49]. Backgrounds vary from uniform hive surfaces to cluttered vegetation, depending on apiary configuration. Traffic density spans at least two orders of magnitude, from sparse early morning activity to intense midday foraging peaks that may exceed hundreds of passages per minute in large colonies during peak bloom [46]. All six evaluated models achieved mAP@50 values of 0.96 or above on the independent test dataset, with inference speeds ranging from approximately 65 to 118 frames per second. The marginal performance differences observed between architecture families (mAP@50 ≤ 0.02) suggest that architecture-specific design choices have limited impact when training data quality and quantity are adequate. Although these benchmarks were obtained on a high-specification GPU workstation, the inference speeds reported in Table 2 confirm that all models exceed the frame rate requirements of standard cameras (25–30 fps). On embedded or edge computing hardware (e.g., NVIDIA Jetson Nano or Raspberry Pi with accelerator), inference speeds are expected to be substantially lower, and lightweight variants such as YOLOv8n or YOLOv11n would be preferable for such deployments. Notably, YOLOv26 variants required substantially longer training times (8.42 h for YOLOv26n and 7.18 h for YOLOv26s) compared to their YOLOv8 and YOLOv11 counterparts, while yielding no meaningful accuracy gain, making them less practical for resource-constrained or real-time field applications. The comparative analysis of YOLOv8n and YOLOv8s further revealed that differences in tracking stability and counting accuracy became more apparent at the video level than at the image level, as detailed in Section 3.

From a methodological standpoint, the performance observed in this study is in line with broader trends in automated biodiversity monitoring, where deep learning approaches increasingly match or exceed expert human performance for species identification and behavioral classification tasks [50,51]. For insects specifically, recent applications have demonstrated successful automated detection and classification in complex field environments, including nocturnal moth monitoring, agricultural pest surveillance, and pollinator or broader insect communities [26,27,52]. Our study extends these advances to the monitoring of social insect behavior by demonstrating that detection and tracking algorithms can be specifically adapted to quantify biologically meaningful individual actions, such as entrance and exit movements. When aggregated over time, these individual-level measurements yield colony-level metrics that are directly relevant to population ecology.

The video-based counting evaluation revealed both the promise and current limitations of the proposed approach. Under low-traffic conditions, the system achieved accurate entrance and exit counts closely matching manual reference values. However, counting accuracy decreased in scenarios involving higher traffic density, frequent occlusions, and rapid movements near the hive entrance. The low counting accuracy observed for specific event types, such as “In” events in Video 1 (34.4%) and “Out” events in Video 3 (45.5%), can be attributed primarily to tracking failures under elevated traffic density and frequent occlusion near the hive entrance. In these scenarios, bees entering or exiting in close succession produced overlapping bounding boxes, causing identity switches and trajectory fragmentation in the BoT-SORT tracker. Additionally, the heterogeneous recording conditions of the publicly available test videos, including variable camera angles, distances, and background complexity, amplified these effects. Practical mitigation strategies include standardizing camera placement to provide an orthogonal view of the entrance board, reducing entrance width to limit simultaneous passage, and employing higher-frame-rate cameras to reduce inter-frame displacement. At the algorithmic level, integrating re-identification modules or appearance-based tracking constraints could reduce identity switches in crowded scenes. These improvements are expected to substantially increase counting accuracy under real operational conditions. Human observers counting fast-moving targets like flying bees exhibit substantial disagreement, particularly during high-traffic periods when error rates may exceed 20% [53]. While the automated system does not yet consistently surpass manual counting accuracy across all conditions, its consistency and the absence of observer fatigue effects suggest that with further refinement of tracking components, automated monitoring has the potential to generate more reliable long-term datasets than those derived from manual observation. This improved measurement precision has direct implications for statistical power in comparative and experimental studies, potentially reducing sample sizes needed to detect biologically meaningful differences in colony activity. The discrepancy between image-level detection accuracy and video-level counting accuracy highlights that tracking stability and temporal trajectory continuity are critical determinants of system-level performance, and that improvements in these areas represent the most promising avenue for enhancing counting reliability. Given the limited number and duration of evaluated video segments, the reported counting results should be interpreted as a proof-of-feasibility demonstration rather than a comprehensive validation of operational counting accuracy.

Beyond its technical performance, the primary biological value of automated entrance monitoring lies in enabling explicit, testable ecological hypotheses. For example, sustained quantification of directional entrance traffic could be used to test whether colonies adjust foraging intensity proportionally to short-term fluctuations in floral resource density, or whether deviations from expected diurnal traffic curves precede measurable declines in brood production or colony weight. Similarly, long-term traffic asymmetries between incoming and outgoing foragers may provide early indicators of stress-related mortality or orientation disruption. By transforming entrance activity into a continuous, high-resolution colony-level behavioral metric, such systems create opportunities to experimentally evaluate causal relationships between environmental change and colony dynamics.

Several ecological applications could benefit from further development and deployment of validated entrance monitoring systems. Colony health assessment represents one priority area, as deviations from expected entrance activity patterns can signal disease, nutritional stress, or exposure to environmental contaminants [12,54,55]. Colonies suffering from pathogens, parasites, or pesticide exposure often exhibit reduced foraging activity, altered diurnal patterns, or abnormal forager mortality, manifesting as asymmetric entrance/exit ratios [56,57]. Continuous monitoring could enable early detection of such anomalies before colony failure becomes irreversible. Similarly, the effectiveness of management interventions (such as supplemental feeding, therapeutic treatments, or queen replacement) can be objectively evaluated by quantifying changes in entrance activity relative to control colonies [19].

Landscape-scale studies of honey bee foraging ecology represent another promising application. By deploying entrance monitors across apiaries distributed along environmental gradients or contrasting land-use contexts, researchers could examine how landscape composition and configuration influence colony-level foraging success [58,59]. Variation in entrance activity among colonies in different landscapes would indicate spatial heterogeneity in resource availability, potentially identifying land management practices that support or limit pollinator populations. When combined with information on floral community composition and bloom phenology, entrance activity data could reveal plant-pollinator interaction dynamics at colony-relevant spatial scales [60].

Climate change impacts on pollinator phenology could also be investigated through entrance monitoring. Long-term datasets on seasonal entrance activity patterns would document shifts in colony spring emergence, foraging season length, and autumn preparation for dormancy [61]. Comparisons across years or geographic locations could identify relationships between temperature trends, floral resource phenology, and colony activity, addressing key uncertainties about how pollinator populations will respond to continued warming [62]. Moreover, extreme weather events (heat waves, cold snaps, droughts, or storms) exert acute effects on honey bee foraging that remain poorly documented due to the difficulty of maintaining observations during such periods. Automated monitoring systems would capture these events without gap, providing data on colony resilience to environmental extremes.

Despite the demonstrated capabilities of our system, several limitations and challenges deserve attention. First, while image-level detection performance was consistently high across all evaluated models, video-based counting accuracy was variable and declined under higher traffic densities. The primary source of counting errors appears to lie in the tracking stage rather than detection, as brief occlusions, rapid direction changes, and overlapping trajectories near the hive entrance can lead to identity switches or missed events. Additionally, the test videos used for counting evaluation were sourced from publicly available online recordings with uncontrolled and heterogeneous capture conditions, rather than from a dedicated, domain-specific video acquisition protocol. Differences in camera angle, resolution, distance to the hive entrance, and background complexity across these videos likely amplified tracking difficulties and contributed to the observed variability in counting performance. Future work employing standardized video recordings captured under controlled, domain-specific conditions is expected to substantially improve counting accuracy and enable more robust system-level validation. Furthermore, expanding training datasets with additional high-density scenarios and exploring more advanced tracking algorithms could further improve robustness in challenging conditions. Second, our current implementation focuses solely on traffic quantification without distinguishing among behavioral states or external indicators such as pollen loads. While entrance counts provide valuable colony-level activity metrics, additional ecological insights could be gained from automated classification of forager behaviors, resource loads, and individual states (e.g., healthy versus diseased) [13,63]. Integrating multi-task learning approaches that simultaneously detect bees and classify their attributes represents a logical extension of our framework, though validation would require larger annotated datasets with fine-grained behavioral labels. Third, while our system operated reliably under the evaluated conditions, during validation trials, long-term deployment in operational settings will require attention to practical considerations, including weather protection for camera hardware, power management for continuous operation, data storage and transmission, and maintenance protocols. Several recent studies have addressed these engineering challenges for camera-based wildlife monitoring, providing relevant precedents for developing robust field deployments [64,65]. Integration with solar power systems and wireless data transmission would enable remote deployment in apiaries lacking electrical infrastructure, expanding the geographic scope of potential monitoring applications. Fourth, our validation focused on entrance activity for *Apis mellifera*, the most widely studied and economically important honey bee species. The model was trained and evaluated exclusively on Western honey bee imagery; its performance on other *Apis* species with different body sizes, coloration, or flight patterns has not been tested and cannot be assumed. Similarly, hive entrance appearance varies considerably across hive types and materials, and performance may differ across these configurations. While the general object detection approach is expected to transfer across taxa and hive types given appropriate retraining data, dedicated validation studies are needed before deployment in non-*mellifera* or non-standard contexts, given the considerable diversity in nesting biology and entrance configurations across bee species [66]. Expanding automated monitoring to diverse wild bee taxa represents an important frontier for pollinator biodiversity research but will require coordinated dataset generation efforts [25]. Therefore, the present results should be interpreted as species- and setup-specific, and not as directly generalizable without retraining and validation under the target conditions. Fifth, we emphasize that entrance activity data, while valuable, represent only one dimension of colony ecology. Comprehensive assessment of colony health and function requires integration with other metrics, including brood production, disease prevalence, nutritional status, and genetic diversity [67,68]. Entrance monitoring is best viewed as a component of multi-faceted monitoring programs rather than a standalone diagnostic tool. Nonetheless, the ease and scalability of automated entrance monitoring relative to more invasive assessment methods (e.g., hive inspections, brood counts, pathogen screening) position it as a valuable first-line surveillance tool that could trigger more detailed investigations when anomalies are detected.

Looking forward, several research priorities emerge from our work. Methodologically, continued refinement of tracking algorithms to handle higher density aggregations and longer tracking durations would expand system capabilities. Integration of behavioral classification modules to automatically identify forager states, interaction events, or unusual activities would enrich the biological information extracted from monitoring data. Development of lighter computational implementations suitable for edge computing devices would reduce power requirements and enable fully autonomous field deployments. From an ecological standpoint, the most pressing need is for longitudinal datasets that leverage automated monitoring to document entrance activity patterns across seasons, years, and environmental gradients. Such datasets would provide unprecedented opportunities to test hypotheses about honey bee population ecology, foraging optimization, colony life history, and responses to anthropogenic environmental change.

## 5. Conclusions

This study demonstrates that automated, image-based detection and tracking can provide accurate identification of individual honey bees and a feasible pipeline for translating these detections into entrance and exit counts at the hive level. Although further refinement of tracking and counting components is required to improve performance under complex field conditions, the proposed framework establishes a robust foundation for continuous, non-invasive colony monitoring. By enabling the transformation of hive entrance activity from an intermittently observed variable into a continuously quantified parameter, this approach supports the assessment of colony dynamics at fine temporal scales. Such advances provide new opportunities for understanding honey bee population processes and offer practical tools for ecological research, precision apiculture, and pollinator conservation.

## Figures and Tables

**Table 1:** Validation, detection performance and computational characteristics of YOLO-based models.

Model	Parameters (M)	Precision	Recall	mAP@50	mAP@50–95	Training Time (h)
YOLOv8n	3.2	0.96	0.95	0.98	0.74	1.44
YOLOv8s	11.2	0.97	0.96	0.99	0.77	5.46
YOLOv11n	2.6	0.96	0.95	0.98	0.74	3.34
YOLOv11s	9.4	0.97	0.96	0.99	0.78	5.45
YOLOv26n	2.4	0.96	0.95	0.98	0.74	8.42
YOLOv26s	9.5	0.97	0.96	0.99	0.78	7.18

**Table 2:** Image-based test detection performance of YOLO-based models.

Model	Precision	Recall	mAP@50	mAP@50–95	F1	Inference (ms/img)	Inference (fps)
YOLOv8n	0.93	0.97	0.96	0.73	0.95	9.78	102.25
YOLOv8s	0.94	0.98	0.98	0.76	0.96	8.45	118.31
YOLOv11n	0.93	0.97	0.97	0.73	0.95	15.32	65.28
YOLOv11s	0.94	0.98	0.98	0.76	0.96	10.27	97.35
YOLOv26n	0.94	0.96	0.96	0.73	0.95	11.67	85.68
YOLOv26s	0.95	0.98	0.97	0.77	0.96	10.61	94.29

**Table 3:** Video-level aggregate entrance–exit counting performance of YOLOv8 models.

Video	Event	Ground Truth	YOLOv8n	YOLOv8s	Accuracy YOLOv8n (%)	Accuracy YOLOv8s (%)
Video1	In	32	11	11	34.4	34.4
Video1	Out	17	12	14	70.6	82.4
Video2	In	4	4	4	100	100
Video2	Out	7	4	5	57.1	71.4
Video3	In	15	19	22	73.3	53.3
Video3	Out	11	17	17	45.5	45.5

## Data Availability

Data are available from the corresponding author upon reasonable request.

## References

[1] Klein A.M., Vaissiere B.E., Cane J.H., Steffan-Dewenter I., Cunningham S.A., Kremen C., Tscharntke T. (2007). Importance of pollinators in changing landscapes for world crops. Proc. Biol. Sci..

[2] Ollerton J., Winfree R., Tarrant S. (2011). How many flowering plants are pollinated by animals?. Oikos.

[3] Gallai N., Salles J.M., Settele J., Vaissière B.E. (2009). Economic valuation of the vulnerability of world agriculture confronted with pollinator decline. Ecol. Econ..

[4] Flores J.M., Gil-Lebrero S., Gamiz V., Rodriguez M.I., Ortiz M.A., Quiles F.J. (2019). Effect of the climate change on honey bee colonies in a temperate Mediterranean zone assessed through remote hive weight monitoring system in conjunction with exhaustive colonies assessment. Sci. Total Environ..

[5] Lemanski N.J., Cook C.N., Ozturk C., Smith B.H., Pinter-Wollman N. (2021). The effect of individual learning on collective foraging in honey bees in differently structured landscapes. Anim. Behav..

[6] Tarpy D.R. (2024). Collective decision-making during reproduction in social insects: A conceptual model for queen supersedure in honey bees (*Apis mellifera*). Curr. Opin. Insect Sci..

[7] Potts S.G., Biesmeijer J.C., Kremen C., Neumann P., Schweiger O., Kunin W.E. (2010). Global pollinator declines: Trends, impacts and drivers. Trends Ecol. Evol..

[8] Vanbergen A.J., Baude M., Biesmeijer J.C., Britton N.F., Brown M.J.F., Brown M., Bryden J., Budge G.E., Bull J.C., Carvell C. (2013). Threats to an ecosystem service: Pressures on pollinators. Front. Ecol. Environ..

[9] Goulson D., Nicholls E., Botias C., Rotheray E.L. (2015). Bee declines driven by combined stress from parasites, pesticides, and lack of flowers. Science.

[10] Lebuhn G., Droege S., Connor E.F., Gemmill-Herren B., Potts S.G., Minckley R.L., Griswold T., Jean R., Kula E., Roubik D.W. (2013). Detecting insect pollinator declines on regional and global scales. Conserv. Biol..

[11] Requier F. (2019). Bee colony health indicators: Synthesis and future directions. CABI Rev..

[12] Meikle W.G., Holst N., Colin T., Weiss M., Carroll M.J., McFrederick Q.S., Barron A.B. (2018). Using within-day hive weight changes to measure environmental effects on honey bee colonies. PLoS ONE.

[13] Jiang J.-A., Wang J.-C., Huang C.-P., Lee M.-H., Liu A.-C., Lin H.-J., Wang C.-H., Chou C.-Y., Yang E.-C. (2024). Foraging flight-based health indicators for honey bee colonies using automatic monitoring systems. Comput. Electron. Agric..

[14] Capela N., Sarmento A., Simoes S., Lopes S., Castro S., da Silva A.A., Alves J., Dupont Y.L., de Graaf D.C., Sousa J.P. (2023). Exploring the External Environmental Drivers of Honey Bee Colony Development. Diversity.

[15] Guzman-Novoa E., Morfin N., Dainat B., Williams G.R., van der Steen J., Correa-Benítez A., Delaplane K.S. (2025). Standard methods to estimate strength parameters, flight activity, comb construction, and fitness of *Apis mellifera* colonies 2.0. J. Apic. Res..

[16] Guezen J.M., Forrest J.R.K. (2021). Seasonality of floral resources in relation to bee activity in agroecosystems. Ecol. Evol..

[17] Polatto L.P., Chaud-Netto J., Alves V.V. (2014). Influence of Abiotic Factors and Floral Resource Availability on Daily Foraging Activity of Bees. J. Insect Behav..

[18] Gary N.E. (1967). A Method for Evaluating Honey Bee Flight Activity at the Hive Entrance1. J. Econ. Entomol..

[19] Meikle W.G., Holst N. (2014). Application of continuous monitoring of honeybee colonies. Apidologie.

[20] Leocadio J.N., Ghilardi-Lopes N.P., Koffler S., Barbieri C., Francoy T.M., Albertini B., Saraiva A.M. (2021). Data Reliability in a Citizen Science Protocol for Monitoring Stingless Bees Flight Activity. Insects.

[21] Crawford E., Leidenberger S., Norrström N., Niklasson M. (2022). Using Video Footage for Observing Honey Bee Behaviour at Hive Entrances. Bee World.

[22] Svec H.J., Ganguly A. (2025). Using AI for Hive Entrance Monitoring. Bee World.

[23] Streit S., Bock F., Pirk C.W., Tautz J. (2003). Automatic life-long monitoring of individual insect behaviour now possible. Zoology.

[24] Quiles-Latorre F.J., Ortiz-López M., Rodriguez-Lozano F.J., Brox M., Flores J.M. (2024). FPGA-Based Bee Counter System. IEEE Access.

[25] Hoye T.T., Arje J., Bjerge K., Hansen O.L.P., Iosifidis A., Leese F., Mann H.M.R., Meissner K., Melvad C., Raitoharju J. (2021). Deep learning and computer vision will transform entomology. Proc. Natl. Acad. Sci. USA.

[26] Schneider S., Taylor G.W., Linquist S., Kremer S.C., O’Hara R.B. (2019). Past, present and future approaches using computer vision for animal re-identification from camera trap data. Methods Ecol. Evol..

[27] Bjerge K., Mann H.M.R., Høye T.T., Sankey T., Ahumada J. (2021). Real-time insect tracking and monitoring with computer vision and deep learning. Remote Sens. Ecol. Conserv..

[28] Bjerge K., Alison J., Dyrmann M., Frigaard C., Mann H., Høye T. (2023). Accurate detection and identification of insects from camera trap images with deep learning. PLoS Sustain. Transform..

[29] Wei M., Zhan W. (2024). YOLO_MRC: A fast and lightweight model for real-time detection and individual counting of Tephritidae pests. Ecol. Inform..

[30] Sledevic T., Serackis A., Matuzevicius D., Plonis D., Vdoviak G. (2025). Visual recognition of honeybee behavior patterns at the hive entrance. PLoS ONE.

[31] Roboflow (2026). Roboflow Universe: Open-Source Datasets for Computer Vision. https://universe.roboflow.com/.

[32] Redmon J., Farhadi A. (2018). YOLOv3: An Incremental Improvement. arXiv.

[33] Ultralytics (2023). YOLOv8 Documentation. https://docs.ultralytics.com/models/yolov8/.

[34] Redmon J., Divvala S., Girshick R., Farhadi A. You Only Look Once: Unified, Real-Time Object Detection. Proceedings of the 2016 IEEE Conference on Computer Vision and Pattern Recognition (CVPR).

[35] Ultralytics (2024). YOLOv11 Documentation. https://docs.ultralytics.com/models/yolo11/.

[36] Ultralytics (2025). YOLO26 Documentation. https://docs.ultralytics.com/models/yolo26/.

[37] Aharon N., Orfaig R., Bobrovsky B.-Z. (2022). BoT-SORT: Robust Associations Multi-Pedestrian Tracking. arXiv.

[38] Eckert C.D., Winston M.L., Ydenberg R.C. (1994). The relationship between population size, amount of brood, and individual foraging behaviour in the honey bee, *Apis mellifera* L. Oecologia.

[39] Khoury D.S., Barron A.B., Myerscough M.R. (2013). Modelling food and population dynamics in honey bee colonies. PLoS ONE.

[40] Holst N., Meikle W.G. (2018). Breakfast Canyon Discovered in Honeybee Hive Weight Curves. Insects.

[41] Woyke J. (1992). Diurnal Flight Activity of African Bees *Apis-Mellifera-Adansonii* in Different Seasons and Zones of Ghana. Apidologie.

[42] Clarke D., Robert D. (2018). Predictive modelling of honey bee foraging activity using local weather conditions. Apidologie.

[43] Kanazoe I.W., Nombré I., Ouédraogo S., Boussim J.I., Vereecken N.J. (2023). Influence of climatic factors and floristic diversity on the foraging activity of Latreille in a West African Savannah. Afr. J. Ecol..

[44] Kulyukin V.A., Coster D., Kulyukin A.V., Meikle W., Weiss M. (2024). Discrete Time Series Forecasting of Hive Weight, In-Hive Temperature, and Hive Entrance Traffic in Non-Invasive Monitoring of Managed Honey Bee Colonies: Part I. Sensors.

[45] Bishop G.A., Fijen T.P.M., Raemakers I., van Kats R.J.M., Kleijn D. (2024). Bees go up, flowers go down: Increased resource limitation from late spring to summer in agricultural landscapes. J. Appl. Ecol..

[46] Mukherjee S., Kulyukin V. (2020). Application of Digital Particle Image Velocimetry to Insect Motion: Measurement of Incoming, Outgoing, and Lateral Honeybee Traffic. Appl. Sci..

[47] Ngo T.N., Rustia D.J.A., Yang E.C., Lin T.T. (2021). Automated monitoring and analyses of honey bee pollen foraging behavior using a deep learning-based imaging system. Comput. Electron. Agric..

[48] Bares J., Poncelet P., Doucet C.M., Legrand C., Cambon A., Carlier C., Chevin P., Dewaele L.P., Jullien D., Thibaud J.B. (2025). Automated identification of honey bee pollen loads for field-applied palynological studies. New Phytol..

[49] Chapman K.E., Smith M.T., Gaston K.J., Hempel de Ibarra N. (2024). Bumblebee nest departures under low light conditions at sunrise and sunset. Biol. Lett..

[50] Wäldchen J., Mäder P., Cooper N. (2018). Machine learning for image based species identification. Methods Ecol. Evol..

[51] Christin S., Hervet É., Lecomte N. (2019). Applications for deep learning in ecology. Methods Ecol. Evol..

[52] Bjerge K., Nielsen J.B., Sepstrup M.V., Helsing-Nielsen F., Hoye T.T. (2021). An Automated Light Trap to Monitor Moths (*Lepidoptera*) Using Computer Vision-Based Tracking and Deep Learning. Sensors.

[53] Odemer R. (2022). Approaches, challenges and recent advances in automated bee counting devices: A review. Ann. Appl. Biol..

[54] Branchiccela B., Castelli L., Corona M., Diaz-Cetti S., Invernizzi C., Martinez de la Escalera G., Mendoza Y., Santos E., Silva C., Zunino P. (2019). Impact of nutritional stress on the honeybee colony health. Sci. Rep..

[55] McKinnon A.C., Collins L., Wood J.L., Murphy N., Franks A.E., Steinbauer M.J. (2023). Precision Monitoring of Honey Bee (Hymenoptera: Apidae) Activity and Pollen Diversity during Pollination to Evaluate Colony Health. Insects.

[56] Gill R.J., Ramos-Rodriguez O., Raine N.E. (2012). Combined pesticide exposure severely affects individual- and colony-level traits in bees. Nature.

[57] Goblirsch M., Huang Z.Y., Spivak M. (2013). Physiological and behavioral changes in honey bees (*Apis mellifera*) induced by *Nosema ceranae* infection. PLoS ONE.

[58] Kennedy C.M., Lonsdorf E., Neel M.C., Williams N.M., Ricketts T.H., Winfree R., Bommarco R., Brittain C., Burley A.L., Cariveau D. (2013). A global quantitative synthesis of local and landscape effects on wild bee pollinators in agroecosystems. Ecol. Lett..

[59] Steffan-Dewenter I., Kuhn A. (2003). Honeybee foraging in differentially structured landscapes. Proc. Biol. Sci..

[60] Cortés-Flores J., Lopezaraiza-Mikel M., de Santiago-Hernández M.H., Martén-Rodríguez S., Cristóbal-Pérez E.J., Aguilar-Aguilar M.J., Balvino-Olvera F.J., Delgado-Carrillo O., Sayago R., Fuchs E.J. (2023). Successional and phenological effects on plant-floral visitor interaction networks of a tropical dry forest. J. Ecol..

[61] Cane J. (2021). Global Warming, Advancing Bloom and Evidence for Pollinator Plasticity from Long-Term Bee Emergence Monitoring. Insects.

[62] de Manincor N., Fisogni A., Rafferty N.E. (2023). Warming of experimental plant-pollinator communities advances phenologies, alters traits, reduces interactions and depresses reproduction. Ecol. Lett..

[63] Marchal P., Buatois A., Kraus S., Klein S., Gomez-Moracho T., Lihoreau M. (2019). Automated monitoring of bee behaviour using connected hives: Towards a computational apidology. Apidologie.

[64] Schneider S., Taylor G., Kremer S. Deep Learning Object Detection Methods for Ecological Camera Trap Data. Proceedings of the 15th Conference on Computer and Robot Vision (CRV).

[65] Weinstein B.G. (2018). A computer vision for animal ecology. J. Anim. Ecol..

[66] Michener C.D. (2007). The Bees of the World.

[67] Delaplane K.S., van der Steen J., Guzman-Novoa E. (2013). Standard methods for estimating strength parameters of colonies. J. Apic. Res..

[68] Eckholm B.J., Huang M.H., Anderson K.E., Mott B.M., DeGrandi-Hoffman G. (2015). Honey bee (*Apis mellifera*) intracolonial genetic diversity influences worker nutritional status. Apidologie.

